# Important Role of Ultrasonography Immediately After Fine-Needle Aspiration Biopsy of Thyroid Nodules to Detect Acute Transient Thyroid Swelling

**DOI:** 10.7759/cureus.67033

**Published:** 2024-08-16

**Authors:** Kouichi Yoshimoto, Shinya Satoh, Hisakazu Shindo, Kento Katsuyama, Daisuke Tatsushima, Takashi Fukuda, Seigo Tachibana, Yusuke Mori, Hiroshi Takahashi, Yuji Nagayama, Hiroyuki Yamashita

**Affiliations:** 1 Department of Surgery, Yamashita Thyroid Hospital, Fukuoka, JPN; 2 Department of Endocrinology, Yamashita Thyroid Hospital, Fukuoka, JPN

**Keywords:** cervical ultrasonography, thyroid tumor, complication, fine needle aspiration biopsy (fnac), acute transient thyroid swelling

## Abstract

Objective: We aimed to determine whether early detection of acute transient thyroid swelling (ATTS) is possible using ultrasonography (US) surveillance immediately after fine-needle aspiration biopsy (FNAB) and discuss the usefulness of routine US after FNAB.

Methods: We retrospectively evaluated the incidence, clinical features, and US and computed tomography findings of ATTS in patients with thyroid nodules who underwent FNABs at our hospital. The study period was divided into two time periods: only symptomatic patients after FNAB were examined using US in the first period (period A: January 2016 to November 2020), whereas all patients were routinely examined using US shortly after FNAB in the second period (period B: December 2020 to December 2022).

Results: We found that the frequency of ATTS increased from 0.18% (10/5,685) in period A to 1.58% (31/1,958) in period B because the majority of ATTS cases in period B were asymptomatic. Follow-up US performed 15 minutes to 3 hours later confirmed no exacerbation of thyroid swelling in patients diagnosed with ATTS during period B. Routine US examinations, shortly after FNAB, significantly reduced the number of return visits after discharge (from 5/10 to 2/31, p=0.006). Furthermore, the incidence of unilateral swelling was higher than previously reported.

Conclusion: Routine US examinations shortly after FNAB may be useful in identifying ATTS regardless of the presence of symptoms; moreover, they may reduce patients’ return visits after leaving the hospital. It is also important to inform patients that delayed complications are possible and that severe cases, although rare, do exist.

## Introduction

Fine-needle aspiration biopsy (FNAB) is the most widely accepted preoperative diagnostic technique for thyroid nodules. It is highly accurate, simple, and cost-effective [1]. This technique is considered to be safe and rarely causes complications. A recent systematic review of thyroid FNAB complications reported pain, hematoma or hemorrhage, vasovagal reactions, vocal cord paralysis, tracheal puncture, and cancer cell spread as major complications [2]. Acute transient thyroid swelling (ATTS), a rare complication of FNAB, is characterized by diffuse thyroid swelling without hemorrhage and is often accompanied by pain [3]. It usually resolves spontaneously but can be fatal, requiring tracheal intubation [4,5] or causing cardiopulmonary arrest [6] owing to airway obstruction. This complication was first reported in the English literature by Haas [7]. To date, approximately 40 cases of ATTS after FNAB have been reported [3-22].

Initially, at our hospital, if ATTS was suspected after FNAB, it was diagnosed using ultrasonography (US) and treated as necessary; however, under this protocol, nearly half of the patients who developed ATTS returned with symptoms of ATTS several hours after leaving the hospital. Therefore, we subsequently changed our protocol to routinely perform US immediately after FNAB to (1) determine if it would change the frequency of ATTS and (2) see if it would reduce the number of patients returning to the hospital after discharge. In this study, we summarized 41 episodes of ATTS after FNAB using the above two protocols and discussed the usefulness of routine US after FNAB. To the best of our knowledge, this has not been reported previously.

## Materials and methods

We performed 7,643 FNABs in patients with thyroid nodules at Yamashita Thyroid Hospital, Fukuoka, Japan between January 2016 and December 2022 (5,685 FNABs in the first period (period A: January 2016 to November 2020) and 1,958 in the second period (period B: December 2020 to December 2022)). US was performed using ALOKA Prosound α7 (Hitachi, Tokyo, Japan) or Aplio i700 (Canon, Tochigi, Japan) with the standardized device settings. The following sonographic characteristics were recorded: size, parenchymal composition, echogenicity, presence or absence of a halo, margin appearance, presence or absence of calcification, and type of calcification. Diagnosis of ATTS was made with the findings of thyroid swelling and hypoechoic cracked lesion, and the disappearance of both findings was used as the criteria for healing.

FNAB was performed under US guidance (parallel approach) using a 22-gauge needle and a 2.5-5 mL syringe. The skin was disinfected with alcohol, and FNAB was performed using an ultrasound gel medium without local anesthesia. The biopsy site was manually compressed immediately after FNAB. In period A, US examination was performed only in symptomatic patients after FNAB, whereas in period B, US was performed approximately five minutes later for all patients, regardless of the presence or absence of symptoms. In both periods, the US was repeated as needed. One patient was on regular anticoagulant therapy during the FNAB.

This study was approved by the Ethics Committee of the Yamashita Thyroid Hospital. This study was conducted as part of our standard clinical practice without intervention, and data were retrieved retrospectively from medical records. Because this was not a clinical trial and the data were anonymized for retrospective analyses, written informed consent was not obtained from the study participants. This single-center, retrospective study was conducted in accordance with the principles of the Declaration of Helsinki.

Serum levels of free T4, thyroid-stimulating hormone, anti-thyroglobulin (anti-TG), and thyroid peroxidase (TPO) autoantibodies were measured using a commercially available ECLusys kit (Roche Diagnostics, Penzberg, Germany).

Data are expressed as means ± standard deviations. Cross-tabulated data were analyzed using Fisher’s exact test. All statistical analyses were performed using JMP software version 17.0 (SAS Institute Inc., Cary, United States). P-values less than 0.05 were considered statistically significant.

## Results

There were 10 (0.18%) and 31 (1.58%) ATTS episodes without significant hematoma formation after FNAB in 10 and 29 patients during periods A and B, respectively. Table 1 summarizes and Table 2 (in the appendix) provides a detailed description of each of the 39 cases with ATTS after FNAB. These patients included one male and nine female patients ranging in age from 34 to 75 years (50.1 ± 11.6) in the A period A and seven male and 22 female patients ranging in age from 27 to 82 years (57.6 ± 15.7) in the B period B. In period A, the thyroid function tests were normal in eight patients and in the subclinical hyperthyroid state in two, whereas in period B, they were normal in 27 patients, in the hypothyroid state in one, and in the subclinical hyperthyroid state in one. Two out of 10 and 6 of 29 patients examined were positive for anti-TG autoantibodies, 2 of 8 and 0 of 19 patients examined were positive for anti-TPO antibodies, and four and six had histories of allergies (due to drugs, foods, bees, or pollen) in periods A and B, respectively.

**Table 1:** Summary of cases with acute transient thyroid swelling following fine-needle aspiration biopsy. ^*^: two patients had ATTS in period A; ^**^: also positive for anti-TG;^ ***^: three cases in periods A and B had two or more symptoms; ^+^: p<0.05; ^++^: p<0.01; ^+++^: p<0.001 ATTS: acute transient thyroid swelling; FNAB: fine-needle aspiration biopsy; TG: thyroglobulin

	Period A	Period B	
Study period	January 2016 to November 2020	December 2020 to December 2022	p-value
fine-needle aspiration biopsy (n)	5685	1,958	
Patients with ATTS (n)	10	29	<0.001^+++^
ATTS episodes (n)	10	31*	<0.001^+++^
The frequency of ATTS (%)	0.18	1.58	<0.001^+++^
Hospital return n (%)	5 (50)	2 (2.2)	<0.01^++^
Men:women (n)	1:9	7.22	0.653
Ages (year) (means ± standard deviation)	50.1±11.6	57.1±15.6	0.206
Thyroid function (n)			
Euthyroid	8	27	0.267
Hypothyroid	0	1	1.000
Hyperthyroid	2	1	0.156
Antibodies (n)			
Anti-thyroglobulin	2	6	1.000
Anti-thyroid peroxidase	0	2**	1.000
Positive for allergies (n)	4	6	0.244
Cases having symptoms after FNAB (n)	10	5	<0.001^+++^
Punctures (n)			
1x	5	12	0.721
2x	5	16	1
3x	0	3	0.556
Symptoms*** (n)			
Uncomfortable feeling	3	2	0.096
Compression feeling	2	0	0.061
Pain	5	3	<0.05^+^
Swallowing difficulty	0	2	1
Cold sweat	0	1	1
Unknown	3	0	<0.05^+^
Time of symptom appearance (n)			
Immediately after	3	2	0.096
An hour later	6	3	<0.01^++^
Not recorded	1	0	0.256
Lobe swelling (n)			
Bilateral	8	20	0.693
Unilateral	2	11	0.445

In period A, all 10 patients had symptoms, such as pain, discomfort, and airway compression, immediately (three patients) or several hours (six patients) after FNAB, and ATTS was confirmed using the US. In period B, 26 of 31 episodes of ATTS did not present any symptoms, two had symptoms such as uncomfortable feeling, cold sweat, and/or pain immediately after FNAB, and ATTS was detected using routine US examination shortly after FNAB. In the other three patients, the thyroid size was normal immediately after FNAB, symptoms developed several hours later, and subsequently, thyroid swelling was observed. Five and two patients in periods A and B, respectively, returned to the hospital because of symptoms after leaving the hospital; the incidence was significantly lower in period B than in period A (p=0.006).

**Figure 1:**
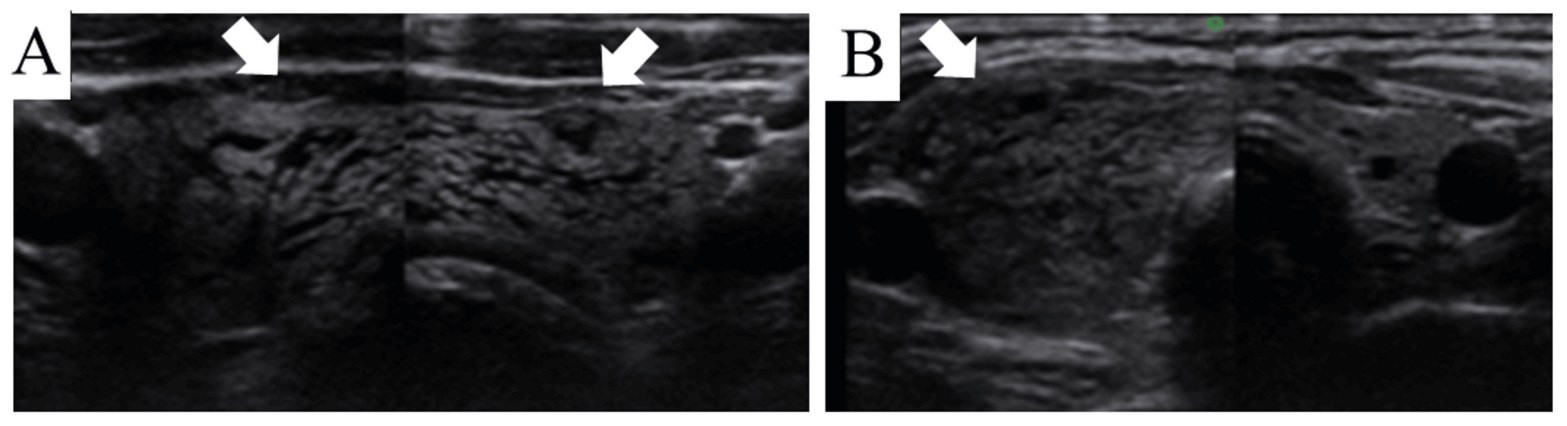
Ultrasound images of the thyroid glands. Bilateral (A) and unilateral (B) thyroid swelling after FNAB with typical ATTS features of hypoechoic cracked lesions. The white arrows depict the swollen thyroid glands. ATTS: acute transient thyroid swelling; FNAB: fine-needle aspiration biopsy

**Figure 2:**
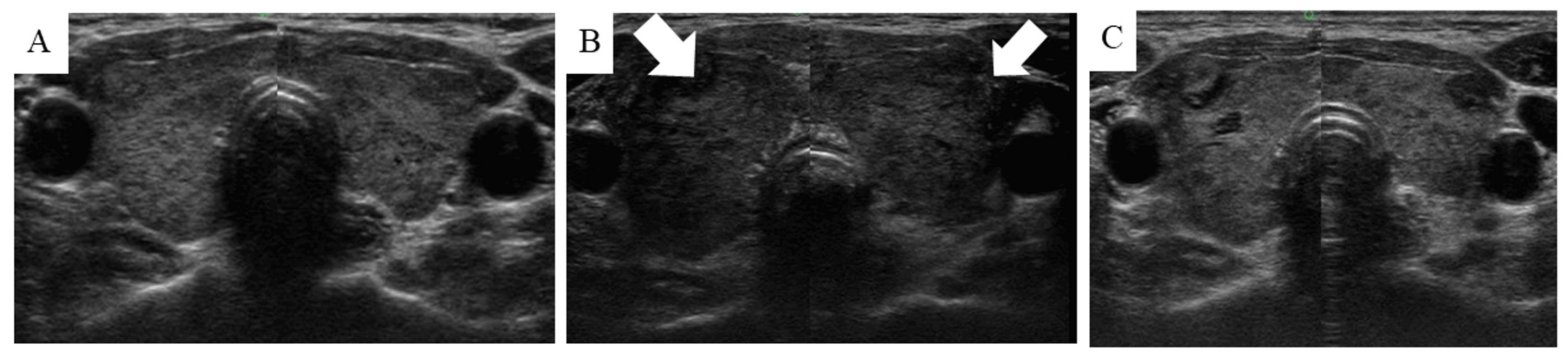
Time course of thyroid swelling. (A) Before FNAB, (B) four hours after FNAB, and (C) two days after FNAB in patient #27. The white arrows depict the swollen thyroid glands. FNAB: fine-needle aspiration biopsy

Swelling developed in both lobes in 28 patients (Figure 1A) and one lobe in 13 patients (Figure 1B); this included the ipsilateral lobes in 10 patients and contralateral sides in two following unilateral biopsies, and one lobe after a bilateral biopsy. Follow-up US performed 15 minutes to 3 hours later showed no worsening of the thyroid swelling. In some cases, the resolution of the swelling was confirmed using the US 1-14 days later (Figure 2). Eight patients (six and two in periods A and B, respectively) were treated with steroids. One patient was hospitalized for one night (patient #2 in period A).

FNAB was repeated in four patients in period B who had ATTS episodes after the first FNAB; two (patients #7 and # 8 in period B) showed similar episodes, and two (patients #10 and #12 in period B) did not after the second FNAB. Conversely, patient #8 in period A did not experience ATTS after the first FNAB but did after the second FNAB.

**Figure 3:**
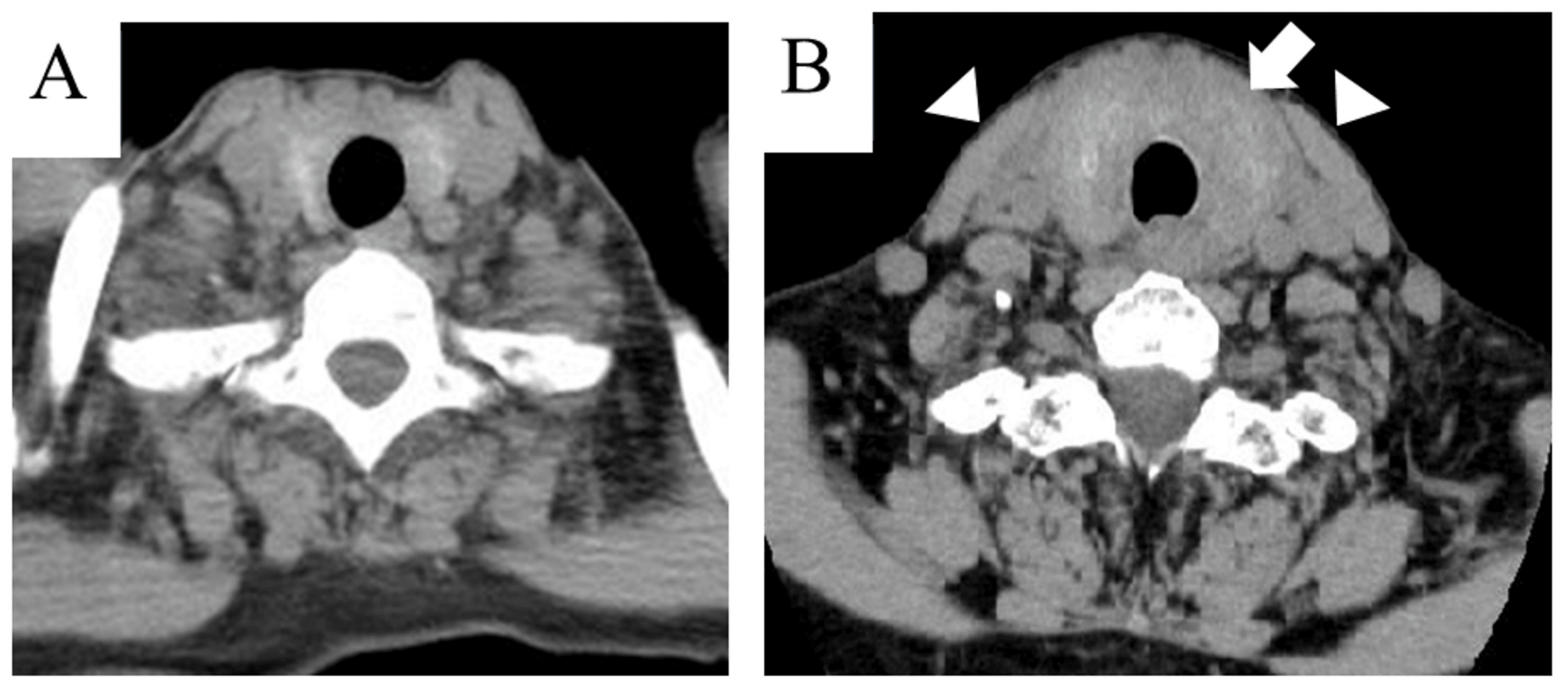
Computed tomography scan. ATTS before (A) and after (B) ATTS in patient #27. The white arrow and arrowheads depict the swollen thyroid and the swollen per-thyroid fat tissues, respectively. ATTS: acute transient thyroid swelling

The US findings were similar to those reported previously; the swollen thyroid glands had a patchy and heterogeneous appearance, with dendritic hypoechoic lesions (hypoechoic “cracks”) scattered throughout (crack-like appearance) in all patients [5]. A computed tomography (CT) scan was performed on two patients and showed increased attenuation of the CT values in the peri-thyroidal fat tissue in addition to diffuse thyroid swelling (Figure 3). A laryngeal endoscopy was performed on six patients and showed no laryngeal edema.

## Discussion

To the best of our knowledge, this is the first study to report an increase in the detection of ATTS, not only by symptomatic presentation but also by US examination five minutes after FNAB, in a relatively large cohort. The incidence rates of ATTS in this study were 0.18% (10/5,685) and 1.58% (31/1,958) in periods A and B, respectively. The observed difference was owing to the initiation of routine US examinations immediately after FNAB in period B, irrespective of the presence of symptoms. In fact, if the patients were restricted to those with symptoms, the frequency dropped to 0.24% (5/1,958) in period B. The frequency of symptomatic ATTS cases in our study was comparable to that reported in previous studies: 0.15% (4/2,742) [15], 0.13% (1/742) [16], and 0.10% (10/9,596) [19].

In most patients in this study, thyroid swelling was detected using the US within 5 minutes, and no exacerbation was confirmed 15 minutes to 3 hours later. The latter is consistent with the previous reports showing spontaneous resolution within 1-20 hours [3]. The patients were successfully managed on an outpatient basis with cooling and compression alone. Steroids were used in eight patients; however, their efficacy was difficult to assess, as previously reported [8]. Thus, the prognosis of ATTS is generally considered favorable. However, we found three recent studies reporting severe cases of airway obstruction owing to FNAB-related ATTS: two patients required tracheal intubation [4,5], and one patient developed cardiopulmonary arrest [6]. Therefore, although some may argue that routine US after FNAB is an excessive test because most ATTS resolve without symptoms, it is important to diagnose ATTS at an early stage using the US, which is non-invasive and rapid. Moreover, the introduction of routine US examinations soon after FNAB significantly reduced the frequency of patients returning to the hospital after discharge. Thus, this intervention may be useful in reducing the number of patients’ return visits after leaving the hospital.

Unilateral swelling occurred in 13 of 41 (32%) patients with unilateral (12 patients) or bilateral (one patient) punctures. This frequency is higher than that in some previous reports (0/10 [19], 0/10 [3], and many others), although unilateral swelling was exceptionally unusually observed in all six patients in one study [14].

Although several hypotheses have been proposed, the exact mechanism underlying ATTS remains unclear. Intrathyroidal edema induced by endogenous substances [10] or idiosyncratic allergic reactions to metallic needles, disinfectants, or US gels [14] have been proposed. A typical US characteristic of ATTS, hypoechoic cracks, may reflect fluid accumulation in the loose interstitial space of the thyroid parenchyma [5,19], which may support both hypotheses (edema and allergy). Typical US findings include inhomogeneous hypoechoic lesions with a crack-like appearance scattered throughout the swollen thyroid glands. However, in two patients in our study, the CT scan also showed swelling of the perithyroidal fat tissue. Two previous studies have reported similar findings: increased attenuation of the perithyroidal adipose tissue, suggesting fluid collection from the retropharyngeal space into the trachea and esophagus in one patient [15], and swelling of the retropharyngeal space requiring tracheal intubation in one patient [5]. In the latter patient, laryngeal fiberscopy revealed protrusion of the posterior pharyngeal wall and edematous changes in the pharyngeal and epiglottal mucosa. These findings indicated the spread of swelling outside the thyroid gland, which could not be detected using the US. Furthermore, the different mechanisms for early- and late-onset cases may be possible.

To date, patients with ATTS have nothing in common regarding their medical history, medications, allergies, sex, age, needle size, use of anesthesia, disinfection fluids, US gel, or US or cytological findings. Moreover, there were no common risk factors of ATTS after FNAB. In our study, there were some patients with and without repeated ATTS, as well as a patient in whom ATTS was absent the first time but developed the second time. Although reports of similar recurrent cases can be found in previous papers [9,14], the exact frequency of recurrence/non-recurrence is unknown. Therefore, it is currently not possible to predict the occurrence of ATTS after FNAB.

Although this study provides valuable information for the management of ATTS, a serious complication after FNAB, it also has a few limitations. These include its retrospective nature, the potential for selection bias, and reliance on US findings without a standardized protocol for diagnosing ATTS and assessing its severity. In addition, the incidence of ATTS is low; therefore, it is difficult to identify clinically significant risk factors for ATTS with the usual statistics, which is why it has not been studied. Thus, we are currently considering a prospective protocol for ATTS after FNAB, as well as its clinical course, including the estimated preprocedural thyroid weight as an objective indicator.

## Conclusions

Our study highlights an improved detection rate of ATTS through routine US examinations performed immediately after FNAB, a practice that has not been previously reported in the literature. Our results suggest that the integration of such US examinations may significantly reduce the need for patients returning owing to late-onset symptoms, thereby improving patient care and reducing hospital revisits. In addition, it is clinically important to inform patients that delayed complications are possible and that severe cases, although extremely rare, do exist.

## Appendices

**Table 2:** Clinical data on individual cases with acute transient thyroid swelling following fine-needle aspiration biopsy. PSL: predonisolone; MPSL: methyl-predonisolone

Case no.	Sex	Age (year)	TSH (μIU/mL)	Anti-thyroglobulin antibody (IU/mL)	Anti-thyroid peroxidase (IU/mL)	Ultrasound diagnosis	Cytology	Biopsied side, no. of puncture	Sites of swelling	Allergy	Treatment	Time of symptom appearance	Symptoms	Follow-up ultrasound
January 2016 to November 2020														
1	F	50	1.42	14.4	Not measured	Adenomatous goiter	Benign	Left, once	Both lobes	None	PSL, antihistaminic	3 hours later (readmission)	Discomfort	Symptom; not exacerbated after 1 hour
2	F	49	1.22	16.2	Not measured	Papillary carcinoma	Papillary carcinoma	Both, one for each	Both lobes	None	MPSL	2 hours later	Pain	Not exacerbated after 1 hour, and symptoms disappeared next morning
3	F	58	0.23	51.1	11.5	Adenomatous goiter	Benign	Both, one for each	Both lobes	Pollen	MPSL, antihistaminic	2.5 hours later (readmission)	Discomfort	Improved after 2 hours, and disappeared 6 days later
4	F	41	2.18	15.4	12.6	Adenomatous goiter	Benign	Left, twice	Both lobes	None	MPSL, antihistaminic	unidentifiable	Compression, pain	Not recorded
5	M	54	1.01	＜10	8.1	Adenomatous goiter	Benign	Left, twice	Both lobes	Unknown	MPSL, antihistaminic	1 hour and 20 minutes later (readmission)	Pain	Improved after 3 hours, and disappeared 14 days later
6	F	34	0.95	28.8	13.5	Adenomatous goiter	Benign	Left, once	Both lobes	None	Cooling and compression	Unknown	Unknown	Not exacerbated 2 days later
7	F	75	0.27	＜10	＜9	Adenomatous goiter	Benign	Left, once	Both lobes	Drug, oyster	MPSL	3 hours and 40 minutes later (readmission)	Compression, pain	Improved after 2 hours
8	F	40	1.19	＜10	13.6	Adenomatous goiter	1st; unsatisfactory 2nd;unsatisfactory	1st; right, twice 2nd; right, once	1st; nothing 2nd; ipsilateral	None	Cooling and compression	Immediately	Compression, pain	Not exacerbated after 1 hour
9	F	44	4.62	＜10	＜9	Follicular tumor	Benign	Right, twice	Ipsilateral	Pollen	Cooling and compression	Immediately	Unknown	Not exacerbated after 15 minutes
10	F	56	1.1	＜10	＜9	Adenomatous goiter	Benign	Left, once	Both lobes	Drug, oyster	Cooling and compression	Immediately	Unknown	Not exacerbated after 45 minutes
December 2020 to December 2022														
1	F	82	0.79	＜10	Not measured	Adenomatous goiter	Indetermined	Right, once	Both lobes	None	Cooling and MPSL	3 hours later	Discomfort, pain, swallowing difficulty	Not exacerbated after MPSL administration (~2 hours)
2	M	67	1.6	＜10	＜9	Adenomatous goiter	Benign	Both, once for each	Both lobes	None	Cooling	Immediately	None	Not exacerbated after 30 minutes
3	F	48	1.47	＜10	＜9	Adenomatous goiter	Follicular tumor	Right, once	Ipsilateral	None	Cooling	Immediately	Discomfort, cold sweat	Disappeared after 20 minutes
4	F	35	1.07	10.2	＜9	Adenomatous goiter	Benign	Left, twice	Contralateral	Shrimp	Cooling	Immediately	None	Slightly exacerbated after 15 minutes, and then shrinking
5	M	56	2.9	＜10	7.8	Adenomatous goiter	Papillary carcinoma	Right, twice	Ipsilateral	Unknown	Cooling and compression	Immediately	None	Shrinking after 20 minutes
6	F	72	1.27	34	Not measured	Adenomatous goiter	Benign	Right, once	Both lobes	Bee	Cooling	Immediately	None	Not exacerbated after 30 minutes
7	F	50	2.93	＜10	15.4	Adenomatous goiter	Benign	1st; right, once 2nd; right, once	1st; ipsilateral, then (15 minutes later) both lobes 2nd; both lobes	None	Cooling	1st & 2nd; immediately	None	Not exacerbated after 15 minutes and 1 hour
8	F	42	1.94	137	42.6	Adenomatous goiter	1st; unsatisfactory 2nd;unsatisfactory	1st; right, once 2nd; right, once	1st; both 2nd; both	None	Cooling	1st & 2nd; immediately	None	Not exacerbated after 1 hour and 30 minutes
9	F	62	0.81	23.5	14.5	Adenomatous goiter	Benign	Left, twice	Ipsilateral	None	Cooling	Immediately	None	Not exacerbated after 30 minutes
10	F	59	1.61	＜10	Not measured	Adenomatous goiter	1st; unsatisfactory 2nd;unsatisfactory	1st; right, twice 2nd; right, twice	1st; both lobes 2nd; None	None	Cooling	Immediately	None	Not exacerbated after 20 minutes
11	M	64	2.46	＜10	＜9	Adenomatous goiter	Indetermined	Right, thrice	Both lobes	None	Cooling	Immediately	None	Shrinking after 20 minutes
12	M	45	0.3L	13	Not measured	Papillary carcinoma	1st; indetermined 2nd; papillary carcinoma	1st; left, once 2nd; left, twice	1st; both lobes 2nd; none	None	Cooling	Immediately	None	Not exacerbated after 20 minutes
13	F	47	2.05	13.4	Not measured	Adenomatous goiter	Benign	Right, once	Both lobes	Pollen	Cooling	Immediately	None	Shrinking after 1 hour
14	M	53	1.73	15	12	Adenomatous goiter	Benign	Left, twice	Ipsilateral	None	Cooling	Immediately	None	Not exacerbated after 20 minutes
15	F	57	1.99	10.6	Not measured	Papillary carcinoma	Unsatisfactory	Right, twice	Both lobes	Pollen	Cooling	Immediately	None	Not exacerbated after 1 hour
16	F	48	1.02	＜10	7.1	Adenomatous goiter	Unsatisfactory	Both, once for right and twice for left	Both lobes	None	Cooling	Immediately	None	Not exacerbated after 20 minutes
17	F	61	4.35	356	25.6	Adenomatous goiter	Unsatisfactory	Right, twice	Both lobes	None	Cooling	Immediately	None	Not exacerbated after 20 minutes
18	F	27	1.72	12.4	＜9	Adenomatous goiter	Medullary cancer	Left, twice	Both lobes	None	Cooling	Immediately	None	Not exacerbated after 1 hour
19	M	35	0.55	11.8	Not measured	Adenomatous goiter	Benign	Right, twice	Both lobes	None	Cooling, compression and PSL	7.5 hours later (readmission)	Swallowing difficulty	Not exacerbated after 1 hour
20	F	73	1.48	192	9.8	Adenomatous goiter	Benign	Right, once	Ipsilateral	Milk	Cooling	Immediately	None	Not exacerbated after 15 minutes
21	F	79	40.6	1180	9.2	Adenomatous goiter	Benign	Right, twice	Ipsilateral	None	Cooling	Immediately	None	Not exacerbated after 30 minutes
22	F	38	1.38	25.6	＜9	Adenomatous goiter	Indetermined	Right, twice	Contralateral	None	Cooling	Immediately	None	Not exacerbated after 30 minutes
23	F	80	2.6	13.5	Not measured	Adenomatous goiter	Unsatisfactory	Right, once	Ipsilateral	None	Cooling	Immediately	Pain	Not exacerbated after 30 minutes
24	F	61	2.28	12.6	＜9	Adenomatous goiter	Benign	Right, twice	Ipsilateral	Loxoprofen, acetoaminofen	Cooling	Immediately	None	Not exacerbated after 20 minutes
25	F	57	1.2	10.8	＜9	Adenomatous goiter	Right: benign; left: unsatisfactory	Both, once for right and twice for left	Left lobe only	None	Cooling	Immediately	None	Shrinking after 20 minutes
26	F	34	1.55	15.7	Not measured	Follicular tumor	Benign	Right, twice	Both lobes	None	Cooling	Immediately	None	Shrinking after 20 minutes
27	F	65	4.65	374	＜9	Adenomatous goiter	Benign	Right, once	Both lobes	None	Cooling	4 hours later (readmission)	Pain	Not exacerbated after 1 hour, and disappeared after 2 days
28	F	82	1.92	12.4	9.3	Adenomatous goiter	Benign	Right, twice	Both lobes	None	Cooling	Immediately	None	Not exacerbated after 50 minutes
29	M	76	1.89	11.9	Not measured	Follicular tumor	Unsatisfactory	Right, twice	Both lobes	None	Cooling	Immediately	None	Not exacerbated after 2 hours
